# Critical Role of Iron in Epoetin Alfa Treatment of Chemotherapy-Associated Anemia

**DOI:** 10.1200/JCO.2016.67.7377

**Published:** 2016-08-08

**Authors:** Irwin Gross, Shannon Farmer, Axel Hofmann, Sherri Ozawa, Aryeh Shander, Matti Aapro

**Affiliations:** Irwin Gross, Eastern Maine Medical Center, Bangor, ME; Accumen, San Diego, CA; Shannon Farmer, University of Western Australia; Curtin University, Perth, Western Australia, Australia; Axel Hofmann, University of Western Australia; Curtin University, Perth, Western Australia, Australia; University of Zurich, Zurich, Switzerland; Sherri Ozawa, Englewood Hospital and Medical Center, Englewood, NJ; Aryeh Shander, Englewood Hospital and Medical Center, Englewood, NJ; Icahn School of Medicine at Mount Sinai, New York, NY; Matti Aapro, IMO Clinique de Genolier, Genolier, Switzerland

## To the Editor:

In the study by Leyland-Jones et al,^1^ we question whether the protocol resulted in adequate iron supplementation in both the epoetin alfa (EPO) group and the best standard of care (BSC) group, but especially in the group that was randomly assigned to receive EPO 40,000 IU once per week. The study design recognized that the availability of iron is a limiting factor in the response to EPO and suggested that, “Patients with transferrin saturation value of less than 20% were to receive iron supplementation”.^1^ There did not seem to be a requirement for iron supplementation after enrollment. We feel the evaluation for functional iron deficiency was inadequate. For patients treated with iron because of baseline transferrin < 20%, iron parameters were remeasured in 3 weeks, which delayed initialization of intravenous iron therapy unnecessarily in patients for whom enteric iron was prescribed. For patients with transferrin > 20%, iron parameters were not remeasured for 6 weeks after random assignment. The authors do not provide the results of iron studies at baseline or during and at the end of study; therefore, we are unable to judge the degree of iron deficiency and functional iron deficiency in the two groups.

That iron and especially intravenous iron was underused in the EPO group is clear from the rate of both enteric and intravenous iron therapy. Oral iron therapy, rather than intravenous iron, was used most frequently (49% of the EPO group and 56% of the BSC group). Intravenous iron use was nearly identical in the two groups (8% in the EPO group and 9% in the BSC group), yet the increased frequency of functional iron deficiency with use of EPO is well known.^2^ The benefit of iron administration in patients with metastatic breast cancer, even when done primarily via oral administration, may explain why, despite chemotherapy, the hemoglobin in both groups rose during the duration of the study from approximately 10.2 g/dL to a median achieved hemoglobin of 11.6 g/dL in the EPO group and 10.9 g/dL in the BSC group. One should also acknowledge that transfusion in the BSC group provides intravenous iron, as donor cells are broken down and heme iron is recycled and used.

Underuse of intravenous iron may have contributed to the higher rate of thrombotic vascular events (TVEs) in the EPO group.^3,4^ EPO can cause functional iron deficiency. Failure to use intravenous iron when administering EPO results in an increased rate of nonresponders and a requirement for higher doses of EPO.^5,6^ Iron deficiency as well as functional iron deficiency is associated with thrombocytosis and increased rates of arterial and venous thrombosis.^7^ Higher target hemoglobin levels are also associated with an increased risk of TVE.^8^ Red cell transfusion is associated with an increased risk of thrombosis.^9^ All patients in the EPO group were treated with EPO to a target hemoglobin of 12 g/dL. Had the BSC group been treated with transfusion to the same hemoglobin target, a higher percentage of patients would have been exposed to the thrombotic risk of red cell transfusion. Risk of TVE in the BSC group is underestimated because a lower target hemoglobin was used. In this study, we think the target hemoglobin in the EPO and BSC groups should have been the same to compare TVE as an adverse outcome in the two groups.

We disagree with the authors’ conclusions that RBC transfusion should be the preferred approach for the management of anemia during first- or second-line chemotherapy for metastatic breast cancer. This recommendation does not take into account the risks of allogeneic red cell transfusion that may be missed in a small study.^10^ The independent review committee–determined primary outcome of progression-free survival met the study criteria of noninferiority. Finally, the difference in TVE as a secondary outcome is likely explained by the higher target hemoglobin in the EPO group, combined with suboptimal iron replacement and EPO-induced functional iron deficiency.
